# Distinct Types of Fibrocyte Can Differentiate from Mononuclear Cells in the Presence and Absence of Serum

**DOI:** 10.1371/journal.pone.0009730

**Published:** 2010-03-18

**Authors:** S. John Curnow, Marianne Fairclough, Caroline Schmutz, Steve Kissane, Alastair K. O. Denniston, Kate Nash, Christopher D. Buckley, Janet M. Lord, Mike Salmon

**Affiliations:** Institute of Biomedical Research, School of Immunity and Infection, College of Medical and Dental Sciences, University of Birmingham, Birmingham, United Kingdom; New York University, United States of America

## Abstract

**Background:**

Fibrocytes are bone-marrow derived cells, expressing both haematopoietic and stromal cell markers, which contribute to tissue repair as well as pathological fibrosis. The differentiation of fibrocytes remains poorly characterised and this has limited understanding of their biology and function. In particular two methods are used to generate fibrocytes *in vitro* that differ fundamentally by the presence or absence of serum.

**Methodology/Principal Findings:**

We show here that fibrocytes grown in the absence of serum (SF) differentiate more efficiently from peripheral blood mononuclear cells than CD14^+^ monocytes, and respond to serum by losing their spindle-shaped fibrocyte morphology. Although fibrocytes generated in the presence of serum (SC) express the same range of markers, they differentiate more efficiently from CD14^+^ monocytes and do not change their morphology in response to serum. Transcriptional analysis revealed that both types of fibrocyte are distinct from each other, fibroblasts and additional monocyte-derived progeny. The gene pathways that differ significantly between SF and SC fibrocytes include those involved in cell migration, immune responses and response to wounding.

**Conclusions/Significance:**

These data show that SF and SC fibrocytes are distinct but related cell types, and suggest that they will play different roles during tissue repair and fibrosis where changes in serum proteins may occur.

## Introduction

Fibrocytes are bone-marrow derived cells that are recruited into sites of tissue injury, most likely as precursor cells, and differentiate into spindle-shaped cells that contribute to repair and angiogenesis, with the potential to induce pathological fibrosis [1], [2]. They are characterised by the expression of both stromal and haematopoietic markers. In particular they produce extracellular matrix molecules including collagen types I and III and fibronectin, while also expressing CD45 and CD34, and can be distinguished from monocytes, macrophages and fibroblasts through a combination of markers [3].

It has been suggested that fibrocytes can act as antigen-presenting cells, responding to TLR ligands [4] and inducing significant T cell proliferation due to the expression of MHC class II and CD86 [5]. However, the primary role of fibrocytes appears to be very similar to local tissue fibroblasts, in providing extracellular matrix deposition during tissue repair, and both cell populations can be isolated from wound chambers [6]. Importantly there is also strong evidence that fibrocytes contribute to pathological fibrosis in a number of tissues, particularly the lung and kidney [7], [8]. Spindle-shaped cells accumulate during bleomycin-induced pulmonary fibrosis where they express both CD45 and collagen I. Fibrosis can be enhanced in this model by intravenous injection of *in vitro*-generated fibrocytes, with the cells recruited to the lungs via their expression of CXCR4 [9]. By contrast, for FITC-induced pulmonary fibrosis, fibrocytes use the CCR2-CCL12 axis for their recruitment [10]. Once fibrocytes have been recruited and/or differentiated in the repairing/fibrotic tissues they may further differentiate towards a myofibroblast phenotype with the expression of α-smooth muscle actin [9], [11]; the precise contribution of fibrocytes to the myofibroblast population may vary depending on the tissue and context. Recently fibrocytes have also been shown to promote healing during colonic inflammation [12].

Fibrocytes can differentiate *in vitro* from either peripheral blood mononuclear cells or purified CD14^+^ monocytes. Thus fibrocytes represent one of a number of cell types that can differentiate from monocytes, including macrophages, osteoclasts and dendritic cells [13]. In the presence of serum, fibrocytes require up to 2 weeks to differentiate [6], whereas in the absence of serum this process is accelerated, with cells appearing in culture after only a few days [14], Interestingly the differentiation of fibrocytes is inhibited by serum amyloid P (SAP), a major constituent of serum [14], and bleomycin-induced pulmonary fibrosis can be inhibited by SAP [15]. A number of other molecules can influence the differentiation of fibrocytes *in vitro*, and presumably *in vivo*. TGFβ1, IL-4 and IL-13 all promote fibrocyte differentiation, with IL-12 and IFNγ being inhibitory [16], [17]. It is currently assumed that fibrocytes that differentiate in the presence or absence of serum reflect the same cell type, differing only in their kinetics of generation. In this study we have addressed this issue by studying the phenotype, growth and differentiation characteristics, response to serum and transcriptome of fibrocytes generated under both serum-free (SF) and serum-containing (SC) conditions. Our data show that these two types of fibrocytes, while closely related, are nevertheless distinct, suggesting that they may play different roles during the response to wounding.

## Results

**Figure 1:**
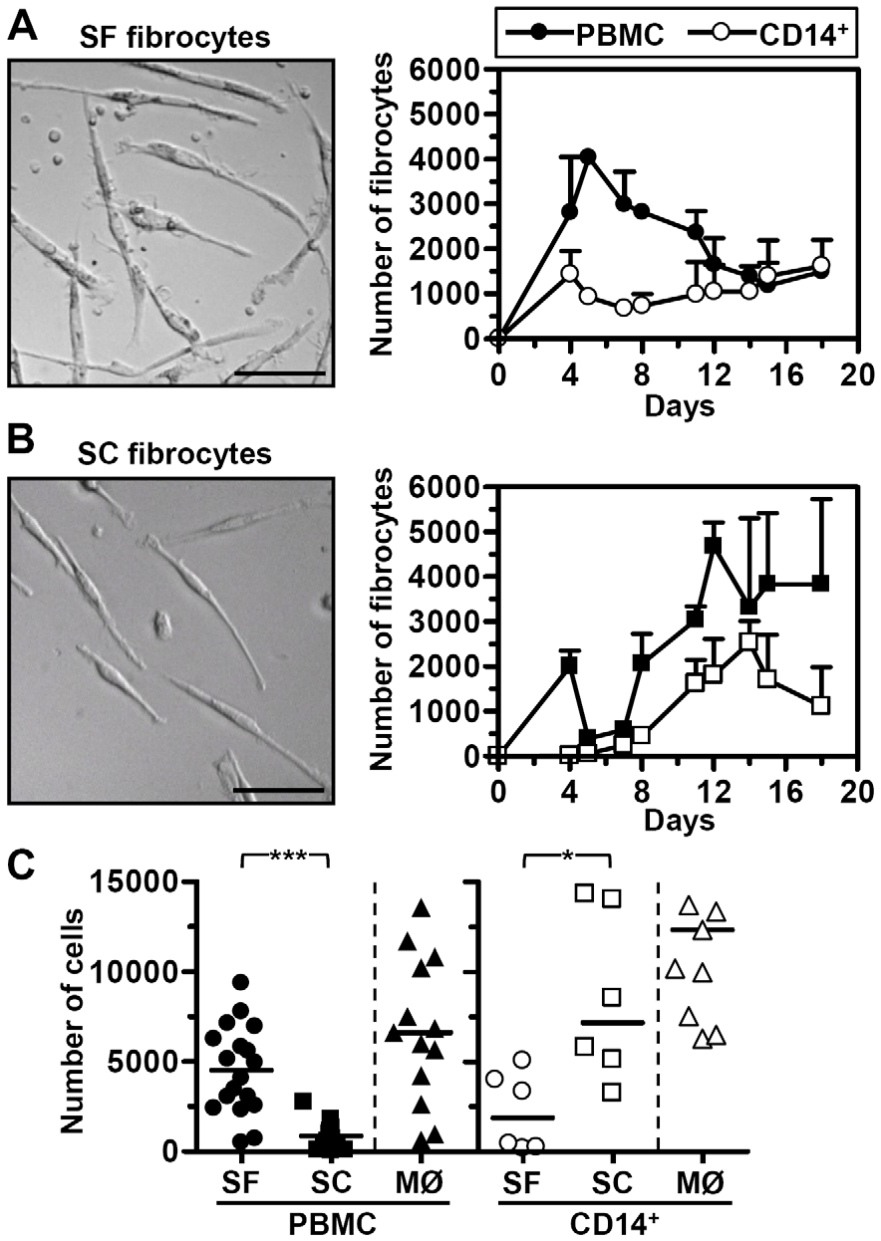
Differential generation of fibrocytes in serum-free or serum-containing conditions, from PBMC and CD14^+^ monocytes. Fibrocytes were generated from PBMC (filled circles) or purified CD14^+^ monocytes (open circles) in either serum-free (SF) or serum-containing (SC; 20% HIFCS). The number of fibrocytes is shown during an 18-day culture period (A,B; mean ± sd of triplicate culture wells). Error bars are only shown in one direction for clarity. Bar represents 50 µm. Fibrocytes and macrophages (MØ) were generated from a number of different normal healthy volunteers (minimum of n = 6) and the number of cells per well counted at day 11 of culture (C). * = p<0.05; *** = p<0.001; Mann-Whitney test.

Fibrocytes were generated by culture of PBMC or CD14^+^ monocytes (purity >98%) in either SF or SC medium (20% heat-inactivated FCS), as previously described in the literature [6], [14]. For both culture conditions, after a few days elongated cells with a fibrocyte morphology began to appear (Fig. 1A,B). In SF conditions the fibrocytes appeared after only 4 days and reached a peak at 6 days, whereas in the presence of serum differentiation required a longer period of culture and did not peak until day 12, as previously described [6], [14]. Interestingly, more SF than SC fibrocytes were generated from PBMC as compared to more SC than SF fibrocytes from purified CD14^+^ monocytes (Fig. 1C). There was no significant difference in the ability of each cell population to generate macrophages. The loss of SC fibrocyte numbers at d5–7 did not occur in all experiments (Fig. S1), and appears to be related to the washing step performed at d4.

**Figure 2:**
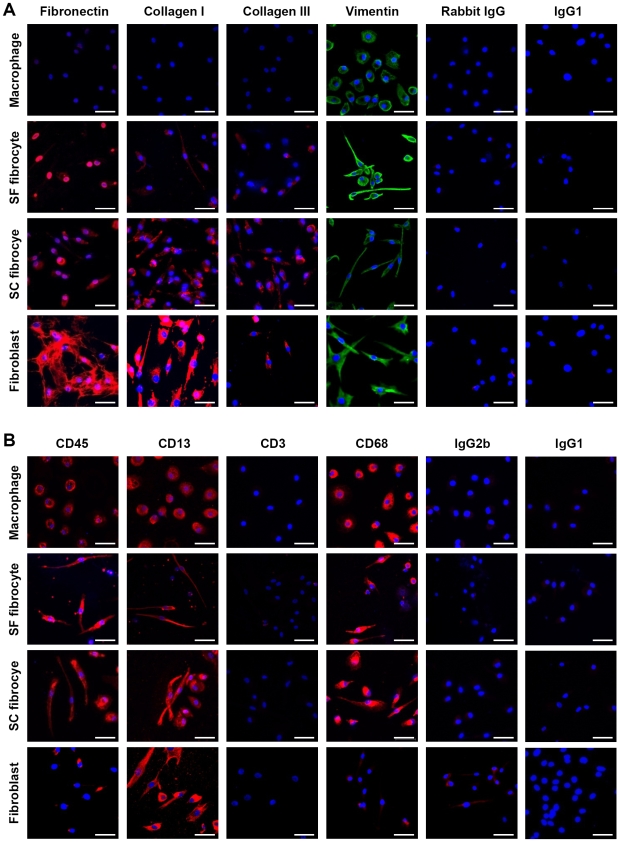
Both serum-free and serum-containing fibrocytes express both haematopoietic and stromal cell markers. Fibrocytes were generated from PBMC under serum-free (SF) and serum-containing (SC) culture conditions, as well as macrophages. Fibroblast cell lines were included as a positive control for stromal cell markers. Immunostaining is shown in red for the stromal markers fibronectin, collagens I and III and their rabbit IgG control, with vimentin and its mouse IgG1 control in green (A). Immunostaining is shown in red for the haematopoietic markers CD45, CD13, CD3 and CD68, and their isotype controls mouse IgG2b and IgG1 (B). Nuclear staining is shown in blue. Bar represents 50 µm. Data are representative of at least two separate experiments.

SF and SC fibrocytes both expressed haematopoietic markers and stromal markers. The stromal markers fibronectin, collagen I and vimentin were expressed by both SF and SC fibrocytes, as well as fibroblasts, but were not expressed by macrophages generated in parallel from the same preparations, except for vimentin which showed a similar degree of expression on macrophages (Fig. 2A). There was also a lower level of expression for collagen III on fibrocytes. The haematopoietic markers CD45 and CD68 were expressed by both types of fibrocyte, as well as macrophages, but were virtually negative on fibroblasts. The few cells that appear to express CD45 are also present with the isotype control. CD13 and CD3 were positive and negative for all cell populations, respectively (Fig. 2B).

**Figure 3:**
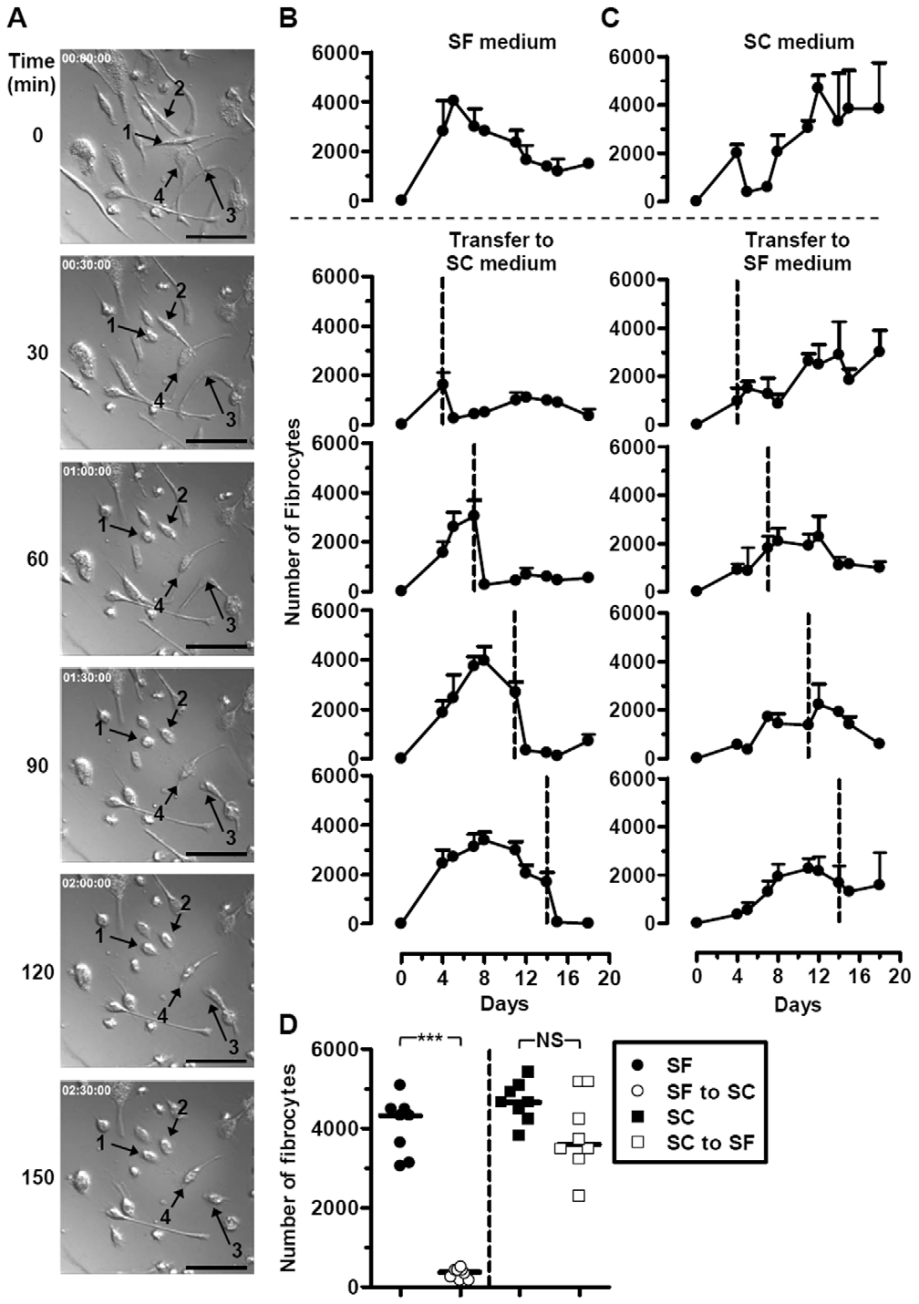
Addition of serum to serum-free generated fibrocytes results in a loss of fibrocyte morphology. Fibrocytes generated from PBMC under serum-free (SF) culture conditions were cultured in the presence of serum-containing (SC) culture medium, and video recordings taken for a period of 150 min. Still photographs are shown after each 30 min period (A), with arrows indicating individual cells that have lost the typical elongated fibrocyte morphology, taking on a more rounded appearance. Bar represents 50 µm. The serum-free culture conditions were changed to serum-containing conditions after 4, 8, 11 and 14 days as indicated by the dotted line (B), with a reciprocal experiment where serum-containing medium was changed to serum-free culture conditions (C). Data are the mean ± sd of triplicate culture wells and are representative of three separate experiments. The median number of cells with fibrocyte morphology at 24 h after the change of culture conditions from multiple experiments is shown (D). *** = p<0.001; NS = not significant, p>0.05; Mann-Whitney test. Error bars are only shown in one direction for clarity.

The differences in the generation of fibrocytes under SF and SC conditions (Fig. 1) suggested that these cells may respond differently to the presence of serum within the culture medium. To investigate the effects of serum on SF fibrocytes we initiated the culture of PBMC in serum-free conditions, and then at either day 4, 8, 11 or 14 replaced the SF medium for SC medium. The number of cells with fibrocyte morphology was assessed until day 18 of the culture. Irrespective of the day at which the serum-containing medium was added there was a loss of cells with a fibrocyte morphology, within 120 min, which persisted for the length of the culture (Fig. 3A,B). Although there was a large, statistically significant, effect of the addition of serum (Fig. 3D), when the reciprocal experiment was performed there was little effect of the change of medium on SC fibrocytes (Fig. 3C,D). Identical results were obtained when cultures were generated from CD14^+^ monocytes (Fig. S1). The effect was found for a variety of different sera, and was not due to the use of RPMI or DMEM culture media (Fig. S2); plasma can also be substituted for serum (data not shown). The expression of both stromal and haematopoietic markers was maintained when SF fibrocytes were switched into SC conditions (Fig. S3), suggesting that the cells were still fibrocytes but had lost their distinct morphology.

**Figure 4:**
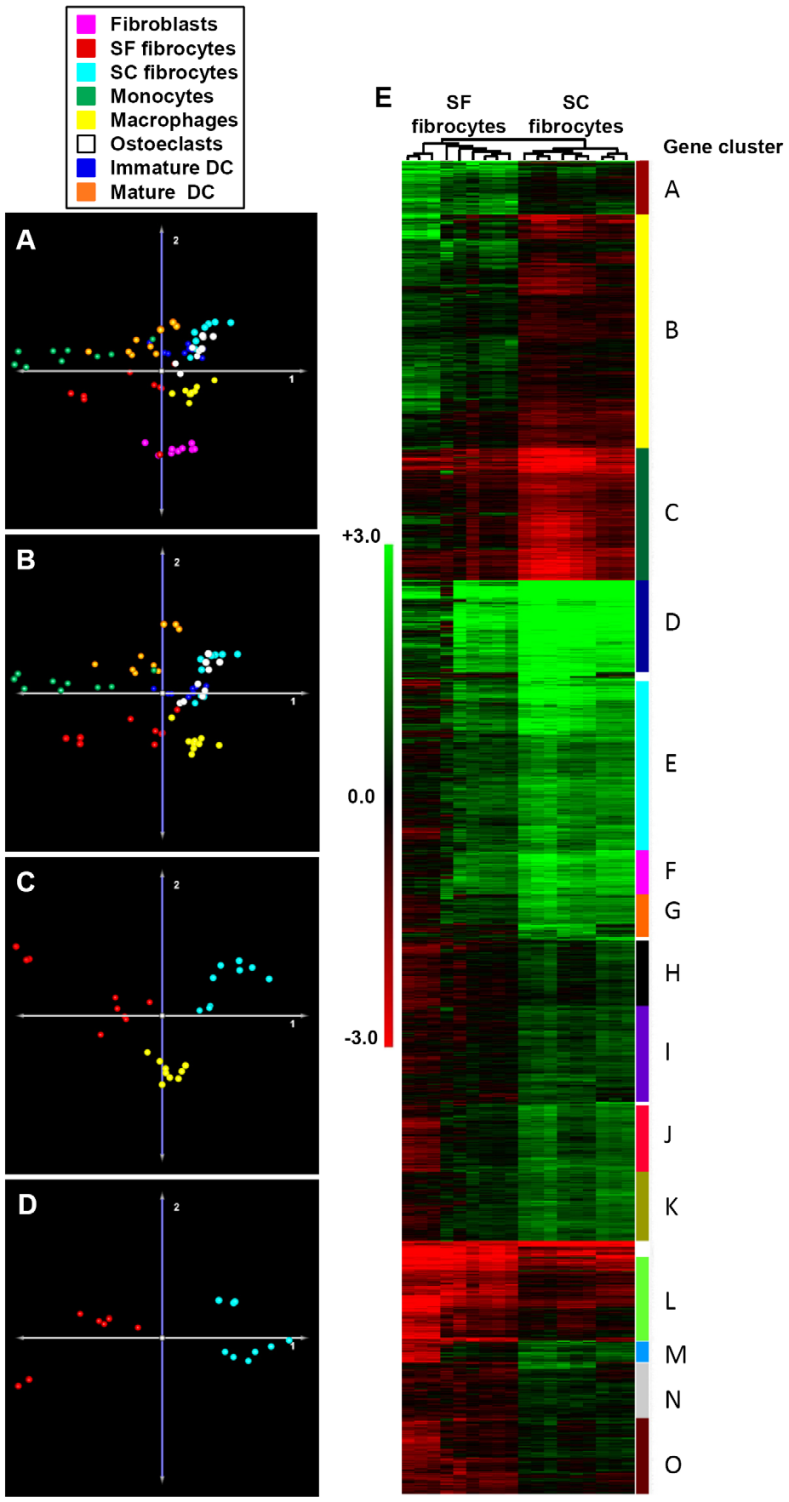
Fibrocytes generated in serum-free and serum-containing conditions show distinct transcriptional profiles from each other, as well as other monocyte-derived progeny. The expression of 35,788 genes was determined by gene microarray analysis for fibroblasts, serum-free (SF) and serum-containing (SC) fibrocytes, monocytes, macrophages, osteoclasts, immature and matured dendritic cells (DC). Data were analysed using significance analysis of microarrays (SAM) for a comparison of all cell populations (A), following removal of the fibroblasts (B), a 3-way analysis of the two fibrocyte populations and macrophages (C) as well as a paired comparison of just the two types of fibrocyte (D). Data are represented as principal component analyses. The SAM gene list was used to perform a hierarchical cluster for both the samples and genes, with each gene cluster labelled (E). The heat map scale represents the gene value relative to a standard reference sample expressed as the log_2_ ratio of reference/sample; therefore green represents higher and red lower relative expression for each sample.

The above data suggested that there were both shared and distinct features of SF and SC fibrocytes. We wished to look more closely at the relationship between these two types of fibrocyte, as well as other monocyte-derived progeny and fibroblasts. cDNA microarrays were performed for each cell population from three different individual donors, each in technical triplicate. The data from SF and SC fibrocytes, CD14^+^ monocytes, immature and mature dendritic cells (DC), osteoclasts, macrophages and fibroblasts were analysed using significance analysis of microarrays (SAM) with a 0.1% false discovery rate, and the resultant gene lists were used for principal component analysis (PCA). When all cell populations were included the SAM identified 24452 significantly different genes (Table S1), and the PCA showed that the fibroblasts formed a distinct cluster away from the other populations (Fig. 4A). Removal of the fibroblasts from the analysis produced a SAM list of 21526 genes (Table S2) and the PCA began to show a distinction between the monocyte-derived progeny (Fig. 4B). In particular the macrophages, SF fibrocytes, monocytes and mature DC formed distinct clusters. Reducing the analysis to just the macrophages, SF and SC fibrocytes generated a list of 3543 genes (Table S3), and the PCA clearly showed three distinct clusters (Fig. 4C). When just the two types of fibrocyte were analysed the SAM list contained 5144 genes (Table S4), and the two cell populations were clearly separated along the first principal component (Fig. 4D). There was always a group of 3 SF fibrocytes that clustered away from the other populations, but still closest to the other SF fibrocytes, and these were triplicates from a single donor. The SAM gene list from the paired 2-way fibrocyte analysis was used to generate a dendrogram, clustered for both type samples and genes. This showed multiple gene clusters that were responsible for the separation of the two types of fibrocyte, with both up and down-regulated genes for each type of fibrocyte. A gene pathway analysis (DAVID) was performed on those genes from the SAM list between the two types of fibrocyte (Table 1; Benjamini p<0.001, >2-fold enrichment). The most significant gene pathways that distinguish SF and SC fibrocytes include the immune and inflammatory response, chemotaxis, RNA processing and lipid metabolism pathways. Genes common to many of the pathways that were up-regulated in SC fibrocytes included TLR4, IL-1β, and the chemokines CCL2, 3, 7 and 22, and the C5a complement receptor. A complete list of the significant DAVID pathways is also provided (Table S5).

**Table 1:** Pathways identified by DAVID analysis of the two-way SAM gene list of serum-free versus serum-containing fibrocytes.

Cluster	Pathway Term	Fold Enrichment^a^	Benjamini
C	GO:0006334∼nucleosome assembly	8.22	8.73E-03
C	GO:0031497∼chromatin assembly	7.89	6.59E-03
D	GO:0002376∼immune system process	2.57	2.40E-05
D	GO:0006955∼immune response	2.69	9.84E-05
D	GO:0006952∼defense response	3.57	1.43E-05
D	GO:0006954∼inflammatory response	3.98	1.74E-03
D	GO:0009605∼response to external stimulus	2.89	1.00E-03
D	GO:0009611∼response to wounding	3.55	5.91E-04
D	GO:0042221∼response to chemical stimulus	2.63	7.46E-03
D	GO:0006935∼chemotaxis	5.36	4.16E-03
D	GO:0007626∼locomotory behaviour	4.7	3.41E-03
D	GO:0042330∼taxis	5.36	4.16E-03
D	GO:0006928∼cell motility	3.16	4.30E-03
D	GO:0051674∼localization of cell	3.16	4.30E-03
D	GO:0006665∼sphingolipid metabolic process	8.72	6.32E-03
D	GO:0030149∼sphingolipid catabolic process	25.57	6.11E-03
D	GO:0006629∼lipid metabolic process	2.52	3.86E-03
D	GO:0046466∼membrane lipid catabolic process	19	3.95E-03
D	GO:0007033∼vacuole organization and biogenesis	19	3.95E-03
D	GO:0006873∼cellular ion homeostasis	4.66	2.04E-03
D	GO:0030003∼cellular cation homeostasis	4.78	4.57E-03
D	GO:0048878∼chemical homeostasis	4.59	2.30E-04
D	GO:0050801∼ion homeostasis	4.23	3.83E-03
D	GO:0055080∼cation homeostasis	4.75	4.61E-03
D	GO:0055082∼cellular chemical homeostasis	4.66	2.04E-03
D	GO:0065008∼regulation of biological quality	2.49	1.93E-03
E	GO:0006464∼protein modification process	2.02	1.34E-07
E	GO:0006512∼ubiquitin cycle	2.93	7.43E-06
E	GO:0043687∼post-translational protein modification	2.08	4.00E-07
E	GO:0008104∼protein localization	2.35	5.60E-05
E	GO:0015031∼protein transport	2.55	1.39E-05
E	GO:0033036∼macromolecule localization	2.21	2.95E-04
E	GO:0045184∼establishment of protein localization	2.44	3.02E-05
J	GO:0015031∼protein transport	3.09	8.70E-03
O	GO:0006396∼RNA processing	5.09	1.23E-06
O	GO:0006397∼mRNA processing	5.83	1.47E-04
O	GO:0008380∼RNA splicing	7.57	1.05E-06
O	GO:0016071∼mRNA metabolic process	5.54	4.45E-05

a The pathways are listed where there was a greater than 2-fold enrichment and a Benjamini-corrected p<0.01, and are grouped according to the gene cluster and biological function.

## Discussion

It is now well established that fibrocytes are bone-marrow derived cells, expressing both haematopoietic and stromal cell markers, that play key roles in tissue repair, angiogenesis and pathological fibrosis [1], [2], [8]. However, two methods have been described in the literature for the *in vitro* generation of fibrocytes from peripheral blood mononuclear cells; the major difference in culture medium is the presence [6] or absence [14] of serum. Given the changes in serum protein concentrations that occur during tissue repair, inflammation and its resolution, understanding the effects of serum on the differentiation and function of fibrocytes is of critical importance. In this study we have compared the growth, phenotype, transcriptome and response to serum of fibrocytes generated under SF and SC conditions. Our data indicate that although both cells can be described as fibrocytes they have distinct properties that may relate to their proposed roles *in vivo*.

The current definition of a fibrocyte is a spindle-shaped cell that expresses both haematopoietic and stromal cell markers. Consistent with published literature [6], [14] both SF and SC fibrocytes expressed CD45 and other haematopoietic markers, as well as fibronectin, collagen I and III and vimentin, both with an elongated spindle-shaped morphology. Therefore, it is clear that the cells differentiated under both SF and SC conditions can be classified as fibrocytes.

Fibrocytes can differentiate *in vitro* directly from CD14^+^ monocytes as well as from a more heterogeneous PBMC population [6], [14], [16]. We observed a difference in the ability of SF and SC fibrocytes to differentiate from PBMC and CD14^+^ peripheral blood cells, with more efficient generation of SF from PBMC, and SC from CD14^+^ cells. This raises the possibility that these cells represent two distinct types of fibrocyte that arise from different precursor cells, present at different frequencies within the PBMC and CD14^+^ cell populations, or differentially affected by the presence of other leukocyte populations. It was previously suggested that T cells enhance the differentiation of SC fibrocytes from CD14^+^ monocytes [16], [18], although paradoxically our data showed a more efficient generation of these fibrocytes from purified CD14^+^ cells than PBMC. We also found a number of other differences between SF and SC fibrocytes. These included the more rapid differentiation of SF fibrocyte in culture, as previously commented on by Pilling et al [14], and distinct transcriptome profiles. Regardless of the origins of SF and SC fibrocytes, our data clearly show that the differentiation in the presence and absence of serum is not identical. Our analysis of the microarray data assumed that any differences observed were due to the fibrocytes and not contaminating monocyte, or monocyte-derived populations. While there is clearly the potential for some contamination we do not believe this to be significant. Although the numbers of fibrocytes in our cultures may appear low compared to the input number, these counts were determined by morphology. The expression of fibronectin (Fig. 2) clearly shows that the percentage of fibrocytes in the cultures was actually very high, and that many cells that would not have been counted as fibrocytes by morphology nonetheless expressed both stromal and haematopoietic markers. In addition, our microarray analysis included both monocytes and monocyte-derived progeny (DC, macrophages and osteoclasts), and yet the fibrocyte populations formed distinct clusters upon analysis of the microarray data.

Monocytes have the potential to differentiate into a number of cell lineages, including macrophages, dendritic cells, fibrocytes, osteoclasts and adipocytes. SAM analysis of the two types of fibrocyte with a number of other monocyte-derived progeny, as well as fibroblasts, revealed that SC fibrocytes were most related to macrophages and osteoclasts, with SF fibrocytes forming a more distinct cluster, away from other monocyte-derived progeny, but also from fibroblasts. The gene pathways identified by DAVID suggest that the SF and SC fibrocytes differ in a number of key areas including response to wounding, inflammatory response and cell migration. Interestingly, a high number of gene pathways associated with lipid metabolism were significantly enhanced in SC fibrocytes. This is consistent with the ability of SC fibrocytes to differentiate to adipocytes [19]. It remains to be determined whether SF fibrocytes retain this degree of multipotency.

Until recently the major cell population thought to be responsible for matrix deposition during tissue repair and fibrosis was the fibroblast. These stromal tissue-resident cells are clearly important players in these processes, with evidence for both their proliferation and differentiation towards myofibroblasts [20]. However, recent evidence has implicated fibrocytes in these processes [1], [2]. During the repair of tissues the concentration of serum-derived proteins will initially be very high, due to the loss of tissue integrity and increased endothelial permeability, and would result in the differentiation of SC fibrocytes. However, as repair proceeds concentrations will fall, favouring the differentiation of serum-free fibrocytes. Although at any one moment in time all infiltrating monocytes would be exposed to the same concentration of serum-derived proteins, this concentration would change during the time-course of the response, and therefore so would the potential to differentiate into either SF or SC fibrocytes. It is possible that the overall serum concentration may control differentiation, or that specific factors present in serum, including serum amyloid P, may drive this process. Although many features were shared between SF and SC fibrocytes (morphology, expression of matrix proteins), the clear transcriptional differences that we observed suggest that they may fulfil distinct roles within the tissue, and that due to the effects of serum on their differentiation this would happen at different times during tissue repair and fibrosis. For example, the differentiation of fibrocytes from monocyte precursors that are constantly recruited to sites of chronic inflammation may favour SF fibrocytes due to the relatively low levels of serum protein that will be present, as compared to acute tissue injury. Interestingly, even when SF fibrocytes had differentiated, we observed a strong effect of serum resulting in the rounding up of these cells. It is possible that this process might allow these fibrocytes to migrate away from the tissue site. We have not been able to identify the component in serum responsible for this change of phenotype, although it does not appear to be SAP (data not shown). This is clearly an important issue for future research, as changes in fibrocyte numbers may either prevent further pathological fibrosis or even contribute to its resolution.

## Materials and Methods

### Ethics statement

This study was approved by the Black Country Research Ethics Committee (07/Q2702/54) and following informed written consent, all samples were collected and stored according to the Human Tissue Act (U.K.).

### Isolation of PBMC and CD14^+^ monocytes

Peripheral blood was taken by venepuncture from healthy volunteer donors into preservative-free heparin. Peripheral blood mononuclear cells (PBMC) were isolated by density-gradient centrifugation using Ficoll-Paque (GE Healthcare Biosciences, Amersham, UK) according to the manufacturer's instructions, and were washed five times with RPMI 1640 to ensure removal of platelets. CD14^+^ monocytes were isolated by positive selection using magnetic beads (Miltenyi Biotec, Bisley, UK). PBMC were re-suspended at 80 µl per 10^7^cells in PBS, 0.5% bovine serum albumin (Sigma-Aldrich, Irvine, U.K.), 2 mM EDTA, with 20 µl of anti-CD14^+^ beads (Miltenyi Biotec) and incubated at 4°C for 15 min. Cells were washed twice by centrifugation before isolation of the bead-bound cells on a magnetised column. A sample of cells were labelled with a FITC anti-CD14 antibody that binds to a distinct (non-competing) epitope from the antibody used for cell isolation (Immunotools, Friesoythe, Germany) and analysed by flow cytometry. Cells were always >95% and routinely >98% CD14^+^ (data not shown).

### Generation of fibrocytes

Fibrocytes were generated by cell culture using two different growth media [6], [14]. Serum-free fibrocyte medium consisted of RPMI 1640 (Sigma-Aldrich), supplemented with 1% GPS (1.64 mM L-glutamine, 40 U/ml benzylpenicillin, 0.4 mg/ml streptomycin; Sigma-Aldrich), 1% Hepes buffer (Sigma-Aldrich), 1% liquid media supplement (ITS+3)(Sigma-Aldrich) 1% non-essential amino acids (Sigma-Aldrich) and 1% sodium pyruvate (Sigma-Aldrich). Serum-containing fibrocyte medium consisted of Dulbecco's Modified Eagle's Medium (DMEM; Sigma-Aldrich) supplemented with 1% GPS and 20% heat-inactivated (HI; 56°C for 1 h) fetal calf serum (FCS; Biosera, Ringmer, U.K.). All cell cultures were performed at 37°C, 5% CO_2_ in a humidified incubator. PBMC (10^6^/ml) or CD14^+^ cells (2×10^5^/ml) were resuspended in either SF or SC medium and cultured in either 8 well glass chamber slides or 24 well plastic plates for the number of days indicated. The starting cell concentrations for each population were those that generated the maximum yield of fibrocytes in preliminary experiments, and also ensured a similar number of monocytes in each. At day 4 the non-adherent cells were washed out and every 7 days half the medium was removed and replaced with fresh medium. The number of fibrocytes was determined by counting triplicate wells with three fields of view per well. Human synovial fibroblast cell lines were generated as previously described [21] and cultured in RPMI 1640, 1% GPS, 10% FCS, 1% non-essential amino acids and 1% ITS+3. Fibroblasts were expanded by trypsin digestion and reseeding into tissue culture flasks of twice the surface area. Cells were harvested for analysis once they had reached confluence, typically after 2-4 days. All fibroblasts were used between passage 3 and 6. Time lapse photography of fibrocyte cultures was carried out using a Zeiss Axiovert 200 and Simple PCI version 5.0 computer software, with a frame every 5 min for at least 6 h. The stage of the microscope was a humidified chamber at 37°C with 5% CO_2_.

### Generation of macrophages, dendritic cells and osteoclasts

In addition to fibrocytes, other monocyte-derived progeny were generated from CD14^+^ monocytes, with additional macrophages generated from PBMC. For macrophage generation, cells were cultured at 10^6^/ml in Iscove's modified Dulbecco's medium (IMDM; Sigma-Aldrich), 1% GPS, 10% pooled human AB^+^ male serum (Biosera) and 50 ng/ml M-CSF (Peprotech EC Ltd., London, UK). Dendritic cells (DC) were generated from 2.5×10^6^ CD14^+^ monocytes resuspended in 5 ml RPMI 1640, 10% pooled human AB^+^ male serum with recombinant human IL-4 (500 U/ml) and GM-CSF (1000 U/ml; Immunotools, Friesoythe, Germany) and cultured for 6 days in T25 flasks (Sarstedt, Leicester, UK). At day 3, 2 ml medium was removed and 2.5 ml fresh medium added. At day 6 immature DC (iDC) were harvested by shaking the flask vigorously and collecting non-adherent cells. To produce mature DC (mDC), harvested iDC were cultured for 2 days with IL-1β (1 µg/ml; Peprotech, London, UK), IL-6 (200 ng/ml; Immunotools), TNFα (10 ng/ml; Peprotech) and PGE_2_ (1 µg/ml; Sigma-Aldrich). Osteoclasts were generated from PBMC by overnight culture in α minimal essential medium (Lonza, Basel, Switzerland), 1% GPS with 10% HI-FCS, followed by removal of non-adherent cells and culture in αMEM, 1%GPS, 10% HI-FCS, with M-CSF (30 ng/ml; Peprotech) and RANKL (25 ng/ml; Peprotech) for 18 days. Medium was replaced with fresh medium every 2 days.

### Confocal immunofluorescence microscopy

Fibrocyte, macrophage and fibroblast cultures were established in 8 well chamber slides and taken to day 11, day 7 and 60% confluence, respectively. Medium was removed, the slides washed three times in PBS for 5 min, air dried and fixed in acetone at −20°C for 15 min. Following rehydration with PBS/2% BSA for 5 min, slides were blocked for 20 min with 10% serum (same species as the secondary antibody used). Following a PBS wash, primary antibodies were added at previously determined optimal concentrations and left overnight in a humidified chamber at 4°C. Following two washes with PBS, secondary antibodies were added and left for one hour. Following two PBS washes, nuclei were counterstained with Hoechst (20 µg/ml; Bis-benzimid H33258 Fluorochrom, Sigma-Aldrich) and slides mounted in 2.4% 1,4-Diazabicyclo[2.2.2]octane (Sigma-Aldrich) in glycerol, pH 8.6. The slides were stored at −20°C until analysis using a Zeiss Axiovert 100M confocal microscope and LSM 5.10 version 3.2 SP2 software. The specific primary antibodies used were rabbit anti-human collagen I (1/100 dilution; Rockland; 600-401-103), collagen III (1/25 dilution; Rockland; 600-401-105) and fibronectin (1/100 dilution; Sigma-Aldrich; F3648), mouse anti-human CD68 (1/25 dilution; BD Pharmingen, Oxford, UK; 556059), CD3 (1/20 dilution; Dako, Ely, UK; M0835), CD13 (1/25 dilution; Immunotools; 21270131), CD45 (1/25 dilution; Immunotools; 21270451) and vimentin (1/100 dilution; Dako; M725) with the isotype-matched irrelevant controls rabbit IgG, mouse IgG2b and mouse IgG1 (1/5 dilution; Dako). The secondary antibodies were Cy5 or Cy3 donkey anti-rabbit (1/125 dilution; Jackson Immunoresearch Labs., West Grove, PA), Alexa Fluor 633 goat anti-mouse IgG1, -mouse IgG2b (1/100 dilution; Invitrogen, Paisley, UK) and FITC goat anti-mouse IgG1 (1/100 dilution; Southern Biotech, Birmingham, Alabama).

### Transcriptome analysis

For transcriptome analysis SF fibrocytes were generated from PBMC and SC from CD14^+^ monocytes, to ensure sufficient numbers from each donor. For each cell type generated triplicate samples were differentiated from each of 3 different donors, with all cell types, except fibroblasts, generated from each donor. Cultures were harvested on days 11, 7, 8, 18 and 100% confluence for fibrocytes, macrophages, iDC/mDC, osteoclasts and fibroblasts, respectively. Microarray slides were scanned using a Scanarray GX+ scanner (Perkin-Elmer, Monza, Italy) and analysed using Scanarray express version 3.03.0002 using variable voltages. A prepared GAL file was used to annotate the slide images and the intensity data was extracted and exported as gpr files using the same software. These were uploaded into the Gene Expression Pattern Analysis Suite (GEPAS) (Bioinformatics Unit CNIO; http://gepas.bioinfo.cnio.es ) software for normalization, which was performed using print-tip loess normalisation with half background subtraction, initially within each slide and slide scale normalisation subsequently between slides. Normalised data were exported to TIGR Multi-experiment Viewer (TMEV), version 4.0 (http://www.tigr.org/tdb/euk ) and hierarchical clustering, significance analysis of microarrays (SAM) and principle component analysis (PCA) were performed. Cluster analysis was performed using average linkage clustering on a Euclidean distance metric matrix using the TMEV data analysis package. Microarray data are available in the ArrayExpress database (www.ebi.ac.uk/arrayexpress) under accession number E-MEXP-2434.

### Online supplemental material

Figure S1 shows the morphology of fibrocytes, differentiated from CD14^+^ monocytes under SF conditions, following the addition of serum. Figure S2 shows the length of fibrocytes, generated under SF conditions, following the addition of serum. Figure S3 shows the expression of vimentin, fibronectin and CD45 following the addition of serum to SF generated fibrocytes. Table S1 is a list of genes identified by SAM (0.1% false discovery rate (FDR)) when comparing monocytes, macrophages, osteoclasts, iDC, mDC, SC fibrocytes, SF fibrocytes and fibroblasts. Table S2 is a list of genes identified by SAM (0.1% FDR) when comparing monocytes, macrophages, osteoclasts, iDC, mDC, SC fibrocytes and SF fibrocytes. Table S3 is a list of genes identified by SAM (0.1% FDR) when comparing macrophages, SC fibrocytes and SF fibrocytes. Table S4 is a list of genes identified by SAM (0.1% FDR) when comparing SC fibrocytes and SF fibrocytes. Table S5 is a list of pathways identified by DAVID analysis of the paired two-way SAM gene list of SF versus SC fibrocytes, where there was a Benjamini-corrected p<0.01 or p<0.0001, and are grouped according to the gene cluster.

## Supporting Information

Figure S1 Addition of serum to serum-free CD14+-generated fibrocytes results in a loss of fibrocyte morphology. Fibrocytes generated from CD14+ monocytes under serum-free (SF) culture conditions were cultured in the presence of serum-containing (SC) culture medium. The serum-free culture conditions were changed to serum-containing conditions after 4, 8, 11 and 14 days as indicated by the dotted line, with a reciprocal experiment where serum-containing medium was changed to serum-free culture conditions. Data are the mean ± sd of triplicate culture wells, and are representative of 3 separate experiments. Error bars are only shown in one direction for clarity.
(0.16 MB TIF)

Figure S2 Decrease in serum-free generated fibrocyte length following addition of serum. A - fibrocytes were generated under serum free culture conditions and the mean length ± sd of 5 fibrocytes is shown calculated from a time-course video of the cells. Fibrocytes were left untreated, or were transferred to medium containing fetal calf serum (FCS) or human serum (HS), with heat-inactivation as indicated (HI). B - the number of cells with fibrocyte morphology is shown (mean ± sd of triplicate culture wells) over a number of days following a change of the culture medium as indicated; serum-free (SF), serum-containing (SC). Error bars are only shown in one direction for clarity.
(0.14 MB TIF)

Figure S3 Serum-free fibrocytes retain expression of stromal and haematopoietic cell markers in the presence of serum. Fibrocytes were generated from PBMC under serum-free (SF) culture conditions and transferred to serum-containing conditions for 24 h before immunostaining for vimentin, fibronectin and CD45. Bar represents 50 µm. Data are representative of two separate experiments. The brightness and contrast of these images have been increased (20% and 25%, respectively).
(0.55 MB TIF)

Table S1 Genes identified by SAM (0.1% FDR) when comparing monocytes, macrophages, osteoclasts, iDC, mDC, SC fibrocytes, SF fibrocytes and fibroblasts.
(34.05 MB XLS)

Table S2 Genes identified by SAM (0.1% FDR) when comparing monocytes, macrophages, osteoclasts, iDC, mDC, SC fibrocytes and SF fibrocytes.
(26.50 MB XLS)

Table S3 Genes identified by SAM (0.1% FDR) when comparing macrophages, SC fibrocytes and SF fibrocytes.
(2.06 MB XLS)

Table S4 Genes identified by SAM (0.1% FDR) when comparing SC fibrocytes and SF fibrocytes.
(2.07 MB XLS)

Table S5 Pathways identified by DAVID analysis of the paired two-way SAM gene list of serum-free versus serum-containing fibrocytes. The pathways are listed for all pathways and those where there was a Benjamini-corrected p<0.01 or p<0.0001, and are grouped according to the gene cluster.
(0.07 MB XLS)
