# Supplementary figures and images for: Distinct Types of Fibrocyte Can Differentiate from Mononuclear Cells in the Presence and Absence of Serum
Source: PLoS One. 2010 Mar 18;5(3):e9730. doi: 10.1371/journal.pone.0009730 (PMC2841180; doi:10.1371/journal.pone.0009730)

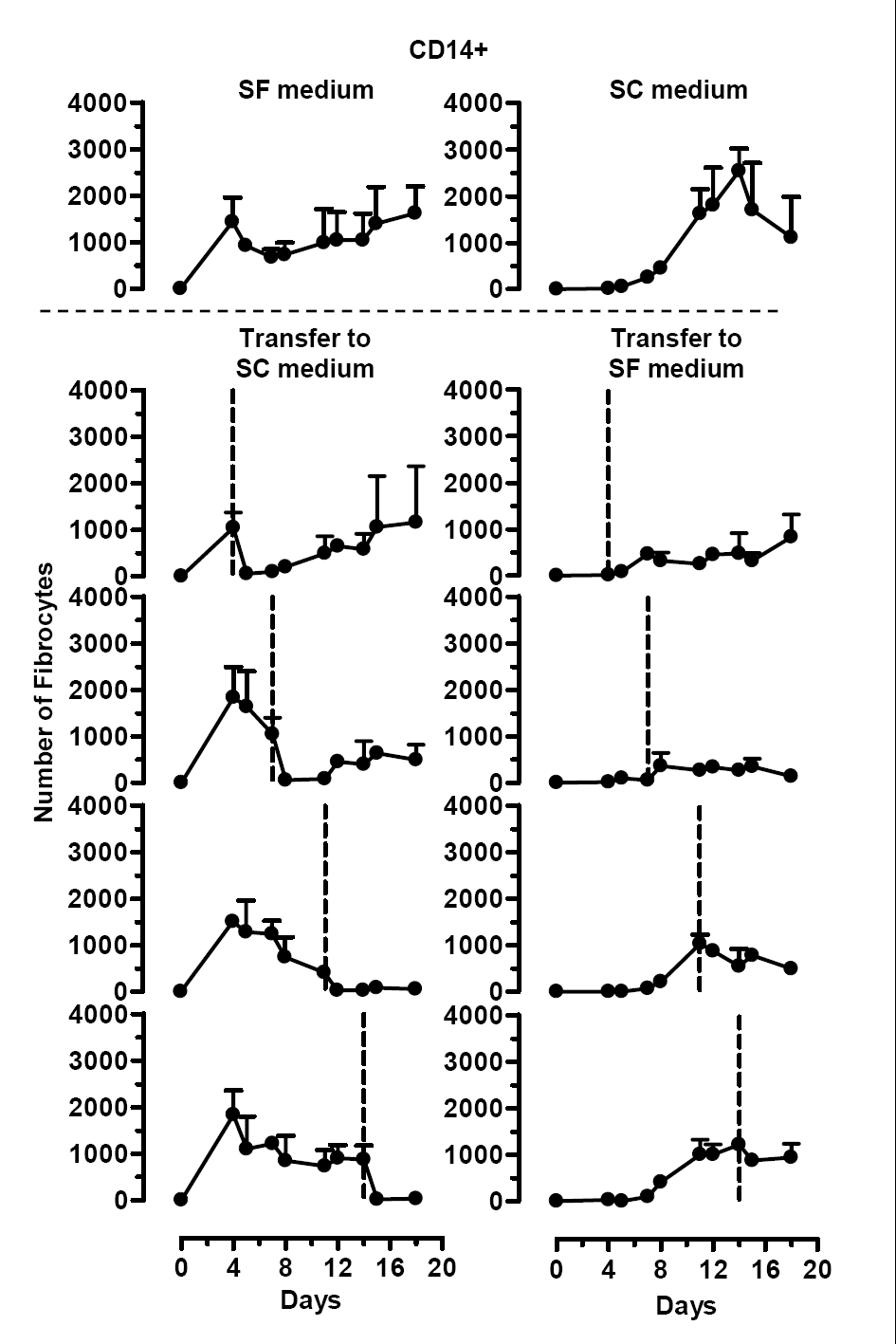

Supplement: Figure S1 — Addition of serum to serum-free CD14+-generated fibrocytes results in a loss of fibrocyte morphology. Fibrocytes generated from CD14+ monocytes under serum-free (SF) culture conditions were cultured in the presence of serum-containing (SC) culture medium. The serum-free culture conditions were changed to serum-containing conditions after 4, 8, 11 and 14 days as indicated by the dotted line, with a reciprocal experiment where serum-containing medium was changed to serum-free culture conditions. Data are the mean ± sd of triplicate culture wells, and are representative of 3 separate experiments. Error bars are only shown in one direction for clarity. (0.16 MB TIF) [file pone.0009730.s001.tif]

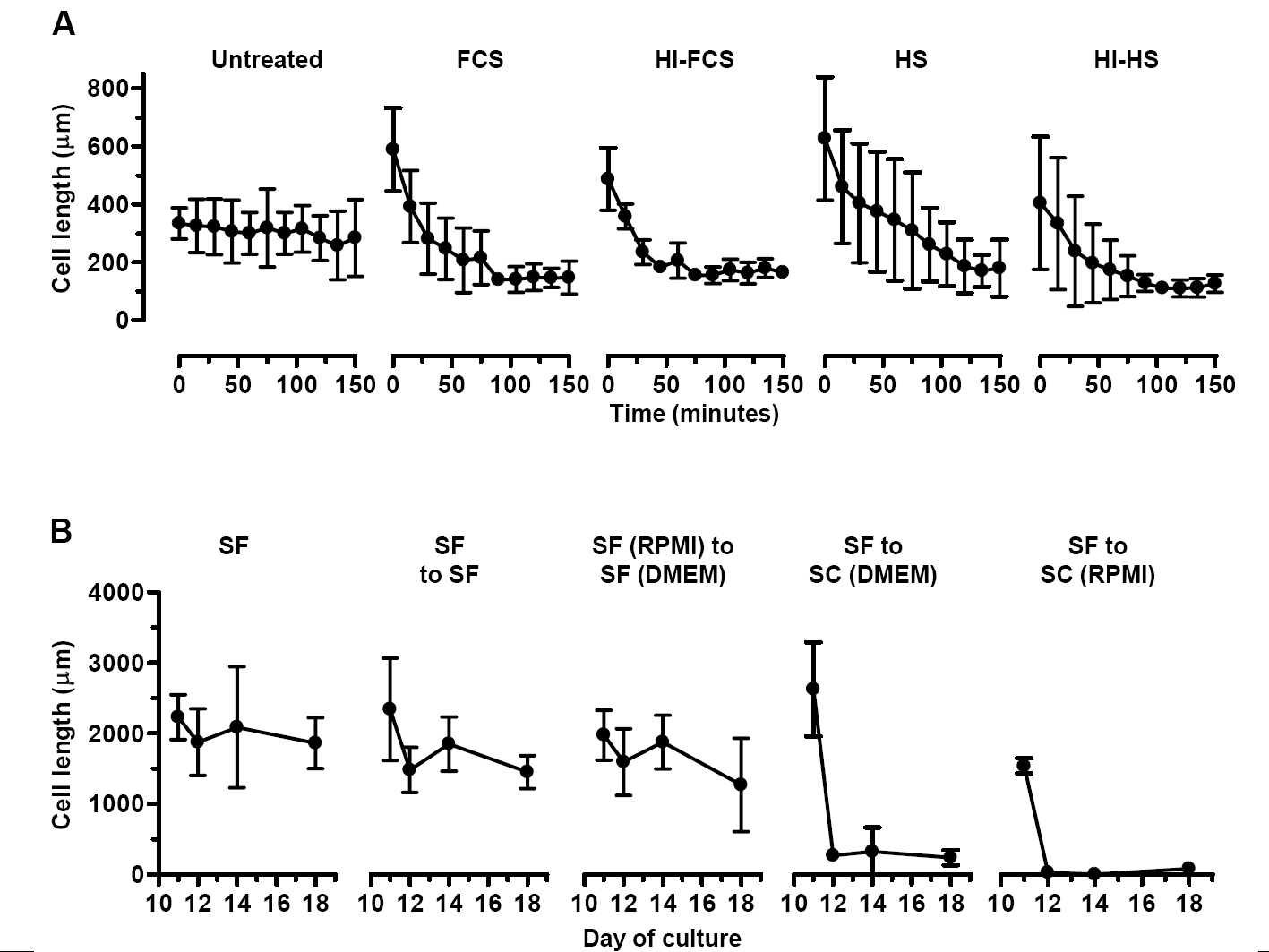

Supplement: Figure S2 — Decrease in serum-free generated fibrocyte length following addition of serum. A - fibrocytes were generated under serum free culture conditions and the mean length ± sd of 5 fibrocytes is shown calculated from a time-course video of the cells. Fibrocytes were left untreated, or were transferred to medium containing fetal calf serum (FCS) or human serum (HS), with heat-inactivation as indicated (HI). B - the number of cells with fibrocyte morphology is shown (mean ± sd of triplicate culture wells) over a number of days following a change of the culture medium as indicated; serum-free (SF), serum-containing (SC). Error bars are only shown in one direction for clarity. (0.14 MB TIF) [file pone.0009730.s002.tif]

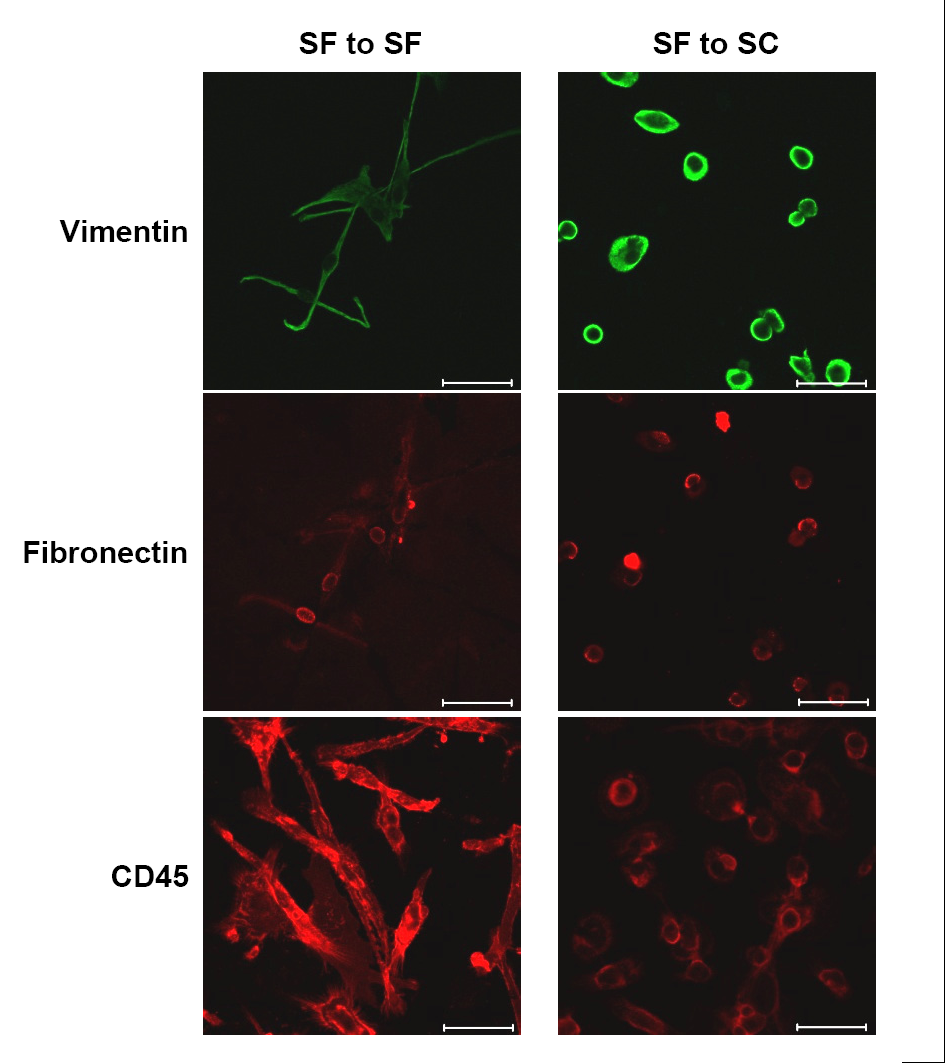

Supplement: Figure S3 — Serum-free fibrocytes retain expression of stromal and haematopoietic cell markers in the presence of serum. Fibrocytes were generated from PBMC under serum-free (SF) culture conditions and transferred to serum-containing conditions for 24 h before immunostaining for vimentin, fibronectin and CD45. Bar represents 50 µm. Data are representative of two separate experiments. The brightness and contrast of these images have been increased (20% and 25%, respectively). (0.55 MB TIF) [file pone.0009730.s003.tif]
